# Water contact angle data and FTIR data of stem cell/tenocyte co-culture-derived secretome releasing electrospun tube; and adhesion data of fully transected and sutured rabbit Achilles tendons three weeks post-operation under application of such a tube

**DOI:** 10.1016/j.dib.2025.112188

**Published:** 2025-10-16

**Authors:** Julia Rieber, Iris Miescher, Petra Wolint, Gabriella Meier-Bürgisser, Jeroen Grigioni, Jess G. Snedeker, Viola Vogel, Pietro Giovanoli, Maurizio Calcagni, Johanna Buschmann

**Affiliations:** a Division of Plastic Surgery and Hand Surgery, University Hospital of Zurich, 8091 Zurich, Switzerland; b Balgrist University Hospital, University of Zurich, 8008 Zurich, Switzerland; c Institute for Biomechanics, ETH Zurich, 8092 Zurich, Switzerland; d Laboratory of Applied Mechanobiology, Department of Health Sciences and Technology, ETH Zurich, 8092 Zurich, Switzerland

**Keywords:** Tendon repair, Fibrotic adhesion, Achilles tendon, Tubular implant, Electrospinning

## Abstract

Tendon ruptures may result in unsatisfactory outcomes, even after long rehabilitation programs and careful return to daily activities and sports. One major problem after a tendon rupture is the fibrotic adhesion formation to the adjacent tissue, which hampers proper motion and may end up in a permanent reduced range of motion.

Following a cell-free approach, we cultivated rabbit adipose-derived stem cells (ASCs) and rabbit Achilles tenocytes in a ratio of 3:1 and incorporated the secretome harvested from this co-culture into an emulsion electrospun tubular DegraPol® fiber mesh. These tubes were characterized by thestatic and dynamic water contact angles and by FTIR spectra, with a comparison to tubes without secretome incorporation (control). Furthermore, we present adhesion data from an in vivo experiment, where these tubes were implanted around a fully transected rabbit Achilles tendon after suture at 3 weeks post-operation.

## Graphical abstract

**Figure:**
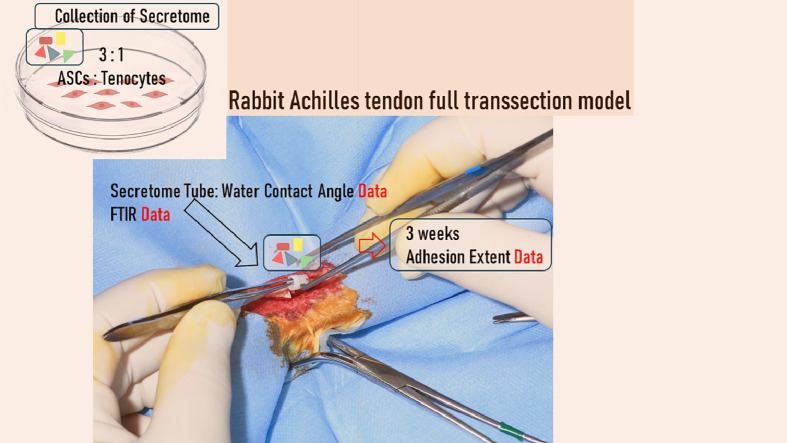

Specifications Table

**Table:** 

Subject	Water contact angle, FTIR, adhesion extent in vivo
Specific subject area	Electrospinning; material characterization; rabbit Achilles tendon in vivo model; fibrotic adhesion
Data format	The data consist of raw and analysed data.
Type of data	Excel files of the type *.xlsx* files.
Data collection	Data collection was performed with different kinds of experiments, including water contact angle (WCA) measurements under static and dynamic conditions; FTIR spectra assessment and histological cross sections of rabbit Achilles tendons to determine the circumference and the parts where the tendons adhered to the surrounding tissue.
Data source location	Except for the WCA and FTIR data, the data were collected in Zurich, Switzerland, at the University Hospital Zurich in the laboratories of the Department for Plastic Surgery and Hand Surgery. The WCA and FTIR measurements were performed at ETH Zurich, Switzerland.
Data accessibility	**Repository name**: Mendeley Data **Data identification number**: 10.17632/x5939mnkxs.1 **Direct URL** to data: Water Contact Angles, FTIR spectra and in vivo Adhesion data (with Micrographs) of Electrospun pure DegraPol® Tubes and of Tubes with Incorporated Secretome Harvested from Rabbit Adipose-Derived Stem Cell/Rabbit Achilles Tenocyte Co-Culture - Mendeley Data
Related research article	Electrospun DegraPol Tube Delivering Stem Cell/Tenocyte Co-Culture-Derived Secretome to Transected Rabbit Achilles Tendon—In Vitro and In Vivo Evaluation Therapeutic Potential of Mesenchymal Stem Cell and Tenocyte Secretomes for Tendon Repair: Proteomic Profiling and Functional Characterization In Vitro and In Ovo

## 1 Value of the Data

• Our data can be reused, if other rabbit cell types than rabbit adipose-derived stem cells or rabbit tenocytes are used to harvest secretome to be incorporated in tubes.
• These data can be reused, if other species than rabbits are used for the extraction of ASCs or tenocytes.
• These data can be compared with other secretomes, particularly with secretomes gathered from pure ASC-culture or pure Achilles tenocyte-culture to elucidate the effect of a co-culture.
• Our adhesion extent data can be used for a comparison with platelet-rich plasma treated rabbit Achilles tendons – also after full transection and repair by conventional suture.

## 2 Background

Different cell-free approaches have been used to support tendon healing after acute injuries; among them, our study using an implant that releases a secretome harvested from a co-culture of ASCs and tenocytes [1]. The problem of fibrotic adhesions during tendon healing is well known, particularly after long times of immobilization [2]. Although early passive and active motion protocols for rehabilitation have gained a lot of attention [3] and may improve the range of motion by mechanically restricting adhesions to the adjacent tissue, biological approaches, targeting cellular pathways, cytokine profiles and/or tissue phenotypes, respectively, have been investigated and reviewed extensively [4]. Among them, especially cell-free approaches using either drugs or growth factors [5] seem to have substantial effects on tendon healing. Finally, approaches affecting epitenon-derived progenitors may be of particular interest, as they play a pivotal role in fibrotic adhesion formation [6].

## 3 Data Description

The data are stored as a set of Microsoft Excel files (Microsoft Corporation, Redmond, WA, USA) (.xlsx files) and a zip folder containing TIFF files (.tif files) in a Mendeley Data repository service Water Contact Angles, FTIR spectra and in vivo Adhesion data (with Micrographs) of Electrospun pure DegraPol® Tubes and of Tubes with Incorporated Secretome Harvested from Rabbit Adipose-Derived Stem Cell/Rabbit Achilles Tenocyte Co-Culture - Mendeley Data.

The following excel files are found in this repository: (i) WCA_Secretome.xlsx; (ii) FTIR_Overview_Secretome.xlsx; (iii) Raw data adhesions secretome in vivo.xlsx and (iv) Adhesion.xlsx, respectively. Furthermore, the following folder is found in the repository: Micrographs.zip.

### 3.1 Water contact angle data

**File** WCA_Secretome.xlsx

This excel file is open access published in Mendeley Data Water Contact Angles, FTIR spectra and in vivo Adhesion data (with Micrographs) of Electrospun pure DegraPol® Tubes and of Tubes with Incorporated Secretome Harvested from Rabbit Adipose-Derived Stem Cell/Rabbit Achilles Tenocyte Co-Culture - Mendeley Data and contains one sheet, called *Tabelle 1*. Within that sheet, column A refers to the sample name; column B to description of the molecular weight of the DegraPol polymer used – either 150 kDa or if nothing is mentioned 80 kDa; column C to where the WCA was measured (either on the outer or the inner surface); column D to Static WCA Replicate 1 (Left); column E refers to Static WCA Replicate 1 (Right); column F to Static WCA Mean Replicate 1; column G to Static WCA Replicate 2 (Left); column H to Static WCA Replicate 2 (Right); column I refers to Static WCA Mean Replicate 2; column J to Static WCA Replicate 3 (Left); column K to Static WCA Replicate 3 (Right); column L to Static WCA Mean Replicate 3; column M refers to the Mean Static WCA; column N to the standard deviation of the Static WCA; column O to Receding WCA (Left); column P to Receding WCA (Right); column Q refers to Mean Receding WCA; column R to the standard deviation of the Receding WCA; column S to indicated means or standard deviations described in the column; column T to Advancing WCA (Left); column U refers to Advancing WCA (Right); column V to Mean Advancing WCA; column W to standard deviation of the Advancing WCA; column X to indicated means or standard deviations described in the column; and column Y refers to hysteresis values calculated as advancing minus receding WCAs.

### 3.2 FITR data

**File** FTIR_Overview_Secretome.xlsx

This excel file is open access published in Mendeley Data Water Contact Angles, FTIR spectra and in vivo Adhesion data (with Micrographs) of Electrospun pure DegraPol® Tubes and of Tubes with Incorporated Secretome Harvested from Rabbit Adipose-Derived Stem Cell/Rabbit Achilles Tenocyte Co-Culture - Mendeley Data and contains 4 sheets, called *Ratio; DP-Tube; Secretome;* and *PEG*, respectively.

The first sheet *Ratio* shows the sample name in column A, the replicate number in column B, the intensity of the *C* = *O* double bond in column C, the intensity of the C—O single bond in column D, and the calculated ratio of *C* = *O* double bond and C—O single bond in column E, respectively. The second sheet entitled *DP-Tube* includes transmission data for DP tube 1 (columns A-C), for DP tube 2_1 (columns d-F) and for DP tube 2_2 (G-I) for each of them 3 replicates, and the mean in column J respectively. The third sheet called *Secretome* shows transmission data for the secretome tube that had a diameter of 3 mm in column A, transmission data for the secretome tube that had a diameter of 4 mm in column B and the calculated mean of these two in column C. The next fourth sheet entitled *PEG* is structured the same as the previous third sheet, however, here, for poly(ethylene glycol) three spectra were taken, with transmission data in column A for PEG 1; in column B for PEG 2 and in column C for PEG 3, while column D presents the calculated mean of columns A-C, respectively.

### 3.3 In vivo application: adhesion extent data in the Rabbit Achilles tendon full transection model

**File** Raw data adhesions secretome in vivo.xlsx

This excel file is open access published in Mendeley Data Water Contact Angles, FTIR spectra and in vivo Adhesion data (with Micrographs) of Electrospun pure DegraPol® Tubes and of Tubes with Incorporated Secretome Harvested from Rabbit Adipose-Derived Stem Cell/Rabbit Achilles Tenocyte Co-Culture - Mendeley Data and contains 1 sheet, called *Raw data adhesion*. In column A, the experimental group and the colour of the rabbit (it was marked with a colour on the ears) is given. In column B, the whole length of the interface between the implant material and the tendon surrounding tissue is given in relative/arbitrary units/entities as assessed by synedra view version 22.0.0.12 software. In columns C-E, adhesion lengths 1, 2 and 3 are shown; depending on if there were different parts of the implant at the interface that adhered to the tendon. In some sections, only one length was measured because it was only one part that adhered. Therefore, there is only one entry under Adhesion length 1. In other sections, several intermittent parts were found to be adhered to the surrounding tissue, so these parts were measured (in arbitrary units/entities) under Adhesion length 1, Adhesion length 2, and Adhesion length 3, respectively. The sum of adhesions is calculated in column F, while in column G the fraction of adhesion is given (sum of all adhesions divided by the whole length). Finally, the fraction is given as a percentage in column H.

**Zip folder** Micrographs.zip

This folder is open access published in Mendeley Data Water Contact Angles, FTIR spectra and in vivo Adhesion data (with Micrographs) of Electrospun pure DegraPol® Tubes and of Tubes with Incorporated Secretome Harvested from Rabbit Adipose-Derived Stem Cell/Rabbit Achilles Tenocyte Co-Culture - Mendeley Data. This zip folder harbors the micrographs referred to in the excel file Raw data adhesions secretome in vivo.xlsx as TIFF files (.tif files).

**File** Adhesion.xlsx

This excel file is open access published in Mendeley Data Water Contact Angles, FTIR spectra and in vivo Adhesion data (with Micrographs) of Electrospun pure DegraPol® Tubes and of Tubes with Incorporated Secretome Harvested from Rabbit Adipose-Derived Stem Cell/Rabbit Achilles Tenocyte Co-Culture - Mendeley Data and contains 1 sheet, called *Adhesion extent 3 weeks*. Column A within this sheet represents the group, with NT = not treated; 4-strand = transected Achilles tendon 4-strand Becker sutured without additional implant; DP = 4-strand Becker suture with a pure DegraPol tube; and Secretome = 4-strand Becker suture, 50 Microliter of a secretome harvested from a 3:1 cell culture of adipose-derived stem cells and rabbit Achilles tenocytes, and a DegraPol tube releasing such secretome applied over the transected and sutured tendon. Column B shows the adhesion extent, calculated as distance of adhered tissue divided by distance of circumference according to a method by Tan et al. [7].

## 4 Experimental Design, Materials and Methods

### 4.1 Water contact angle (WCA) – static and dynamic

The WCA was assessed for DP tubes and DP with secretome tubes (*n* = 3). All tubes’ surfaces were measured on the inner and outer layers [1]. The static WCA was determined by a goniometer equipped with an IDS uEye camera. Five microliters of Milli-Q water were placed on the sample surface with a 1 ml syringe. The left and right WCA were recorded with *n* = 3 repetitions for each assessment.

**Fig. 1:**
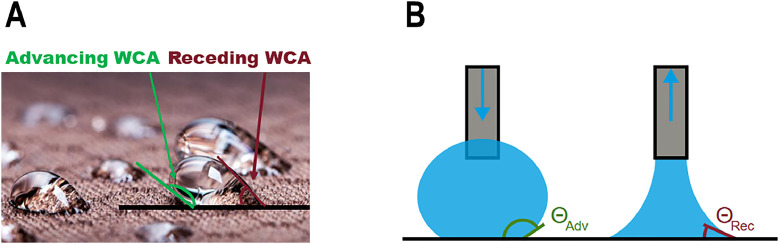
Scheme to show advancing (Adv) and receding (Rec) water contact angles for water droplets on a surface (**A**) and for the measurements (**B**). Images according to *Dataphysics* (Understanding Interfaces) Dynamic contact angles explained - DataPhysics Instruments.

Also dynamic WCA were measured with this setup. The water was either added or withdrawn at a specific speed of 15 microliters per minute to measure the advancing and receding WCAs (Fig. 1), respectively, in intervals of one second. The hysteresis was calculated as follows: the advancing angle minus the receding angle [1].

### 4.2 Fourier transformed infrared spectroscopy FTIR

The FTIR spectra were assessed on a Varian 640 FTIR spectrometer equipped with a Golden Gate diamond ATR unit (*n* = 3 tubes per condition). The ratio of the C=O peak intensity at 1720 cm⁻¹ to the C – O peak intensity at 1175 cm⁻¹ was calculated. All spectra were normalized relative to the C=O peak at 1720 cm⁻¹ [1].

### 4.3 In vivo experiments to assess adhesion extent

**Fig. 2:**
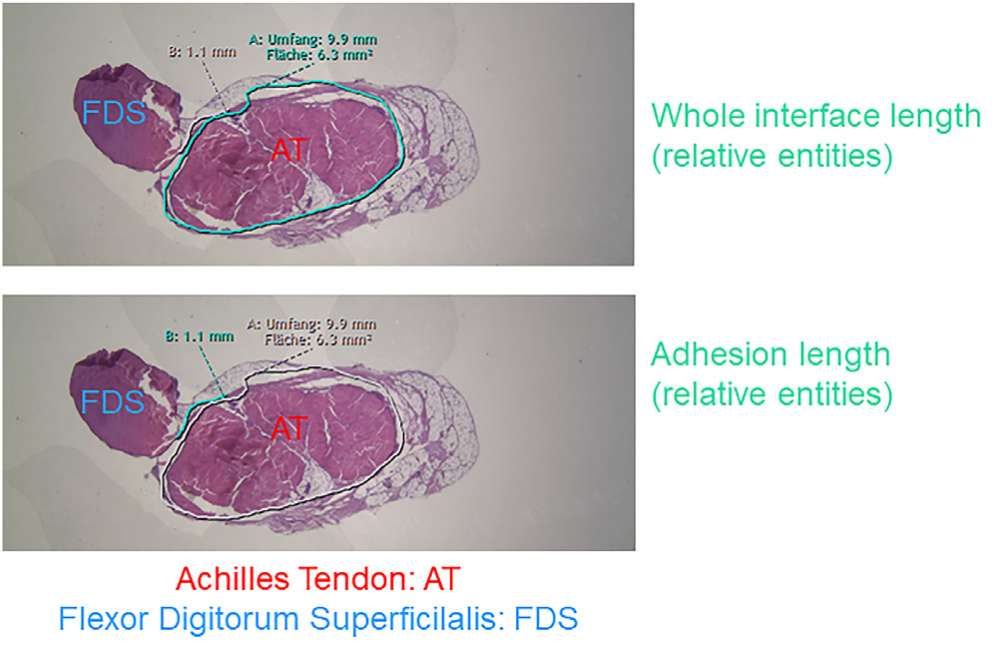
Example of an H&E stained cross section of a rabbit Achilles tendon (AT) and the flexor digitorum superficialis (FDS). With synedra view version 22.0.0.12 software, the whole length of the interface between tendon and surrounding tissue was marked and measured (upper image); as well as the adhesion length (lower image), utilizing relative entities. The percentage of adhered tissue was calculated as adhesion length divided by whole length according to a method from Tan et al. [7].

All rabbit Achilles tenotomies and sutures were performed as previously reported for this preclinical acute tendon injury model [8]. After three weeks in vivo, tendons were extracted and cross sections were stained with Hematoxylin-Eosin (H&E). The whole length between the Achilles tendon and the surrounding tissue as well as the contact region (= adhesion length) was measured by synedra view software (version 22.0.0.12) in relative entities, before dividing the length of the contact region by the length of the whole length to yield the adhesion extent, following a protocol by Tan et al. [7] (Fig. 2).

## Limitations

The in vivo experiments were performed with an endpoint at three weeks. Longer periods would be highly interesting, because adhesion formation to the adjacent tissue is known to develop until approximately six weeks post-operation. Furthermore, the rabbit Achilles tendon is not an intrasynovial tendon model; however, particularly intrasynovial flexor tendons of the hand are prone to develop severe adhesions. Therefore, this study is limited to Achilles tendons, where adhesions may occur, but are not such a pronounced problem as encountered in hand tendons.

## Ethics Statement

The study was conducted in accordance with the veterinary office of the Canton Zurich, Switzerland, with the ethical approval under licence No ZH 080/ 2021.

## CRediT Author Statement

**Julia Rieber:** Conceptualization, Methodology, Data curation, Investigation, Writing – Original draft preparation, Writing – Reviewing and Editing**. Iris Miescher**: Conceptualization, Methodology, Data curation, Investigation, Writing – Reviewing and Editing. **Petra Wolint**: Conceptualization, Methodology, Data curation, Investigation, Writing – Reviewing and Editing. **Gabriella Meier Bürgisser**: Data analysis, Writing – Reviewing and Editing. **Jeroen Grigioni**: Data analysis, Writing – Reviewing and Editing. **Jess G. Snedeker**: Supervision, Writing – Reviewing and Editing. **Viola Vogel:** Supervision, Writing – Reviewing and Editing. **Pietro Giovanoli**: Supervision, Writing – Reviewing and Editing. **Maurizio Calcagni**: Supervision, Writing – Reviewing and Editing. **Johanna Buschmann**: Conceptualization, Supervision, Writing – Original draft preparation, Writing – Reviewing and Editing, Fund raising, Administration.

## Data Availability

(Mendeley Data).Water Contact Angles, FTIR spectra and in vivo Adhesion data (with Micrographs) of Electrospun pure DegraPol® Tubes and of Tubes with Incorporated Secretome Harvested from Rabbit Adipose-Derived Stem (Original data)

## References

[1] Rieber J., Miescher I., Wolint P., Bürgisser G.M., Grigioni J., Snedeker J.G., Vogel V., Giovanoli P., Calcagni M., Buschmann J. (2025). Electrospun DegraPol tube delivering stem cell/tenocyte Co-culture-derived secretome to transected rabbit achilles tendon—In vitro and In vivo evaluation. Int. J. Mol. Sci..

[2] Graham J.G., Wang M.L., Rivlin M., Beredjiklian P.K. (2019). Biologic and mechanical aspects of tendon fibrosis after injury and repair. Connect. Tissue Res..

[3] Shaw A.V., Verma Y., Tucker S., Jain A., Furniss D. (2023). Relative motion orthoses for early active motion after finger extensor and flexor tendon repairs: a systematic review. J. Hand Ther..

[4] Najafi Z., Rahmanian-Devin P., Baradaran Rahimi V., Nokhodchi A., Askari V.R. (2024). Challenges and opportunities of medicines for treating tendon inflammation and fibrosis: a comprehensive and mechanistic review. Fundam. Clin. Pharmacol..

[5] Rieber J., Wolint P., Meier-Bürgisser G., Ongini E., Giovanoli P., Calcagni M., Snedeker J.G., Buschmann J. (2025). Synergistic effects of insulin-like growth factor-1 and platelet-derived growth factor-BB in tendon healing. Int. J. Mol. Sci..

[6] Nichols A.E.C., Benoodt L., Adjei-Sowah E., Jerreld K., Kollar A., Ketonis C., Loiselle A.E. (2025). Epitenon-derived progenitors drive fibrosis and regeneration after flexor tendon injury in a spatially-dependent manner. Nat. Commun..

[7] Tan V., Nourbakhsh A., Capo J., Cottrell J.A., Meyenhofer M., O'Connor J.P. (2010). Effects of nonsteroidal anti-inflammatory drugs on flexor tendon adhesion. J. Hand Surg. Am..

[8] Meier Buergisser G.M., Calcagni M., Bachmann E., Fessel G., Snedeker J.G., Giovanoli P., Buschmann J. (2016). Rabbit Achilles tendon full transection model - wound healing, adhesion formation and biomechanics at 3, 6 and 12 weeks post-surgery. Biol. Open.

